# Comprehensive evaluation of current strategies in achalasia treatment: Insights from an umbrella review

**DOI:** 10.1097/MD.0000000000045885

**Published:** 2026-05-12

**Authors:** Negin Letafatkar, Arman Habibi, Fatemeh Mohammadyari, Farahnaz Joukar, Sara Nobakht, Seyed-Kazem Hosseini-Ghaziani, Mohammad-Hossein Keivanlou, Ehsan Amini-Salehi, Soheil Hassanipour, Fariborz Mansour-Ghanaei

**Affiliations:** a Gastrointestinal and Liver Disease Research Center, Guilan University of Medical Sciences, Rasht, Iran.

**Keywords:** achalasia, botulinum toxin, laparoscopic Heller myotomy, overview review, peroral endoscopic myotomy (POEM), pneumatic dilatation, umbrella review

## Abstract

**Background:**

Achalasia poses a considerable clinical challenge due to its complex pathophysiology and varied treatment responses. This umbrella review aims to consolidate current meta-analyses, providing a comprehensive evaluation of treatment efficacy across various methodologies. By employing statistical analyses and concise quality assessment, we seek to standardize outcomes and effectively highlight the limitations inherent in existing research.

**Methods:**

A comprehensive literature search was conducted across 5 international databases, including PubMed, Scopus, Web of Science, Embase, and the Cochrane Library, to identify relevant meta-analyses focused on achalasia treatments. Key parameters evaluated included clinical success rates, complications, operation times, and lengths of hospital stay. Statistical synthesis was employed to compare treatment effectiveness across studies.

**Results:**

The initial search identified 1037 records, of which 23 meta-analysis studies were included in this study. This review suggests that peroral endoscopic myotomy (POEM) may be comparable to, or potentially more effective than, Heller myotomy in the short-term management of achalasia. However, this was not further supported in longer follow-up durations. While POEM offers advantages such as shorter hospital stays, the ability to perform longer myotomies, and reduced operative duration, it demonstrated a greater risk of gastroesophageal reflux. However, the existing data are insufficiently conclusive, underscoring the need for additional long-term, high-quality studies to validate these results.

**Conclusion:**

This review highlights the comparable efficacy and safety of POEM and Heller myotomy in the treatment of achalasia. The variability in study quality and the presence of methodological flaws in the conducted studies underlines the need for further long-term research, improved methodological rigor, and standardized treatment protocols to optimize patient management in achalasia.

## 1. Introduction

Achalasia, a rare primary esophageal disorder, is caused by neurodegeneration that affects the myoenteric plexus of the esophagus, the lower esophageal sphincter, the vagal trunks, and the dorsal vagal nucleus.^[1]^ Food stasis and impeded bolus transit in the esophagus are the results of this irreversible functional impairment.^[2,3]^ The main pathogenic mechanisms of achalasia are not clear, although possible causes include drug-induced, autoimmune, idiopathic, or genetic factors. Infections like chronic Chagas disease, which is most associated with *Trypanosoma cruzi* infections, may also be to blame.^[4,5]^ Achalasia appears as a steady progression over years, with symptoms including dysphagia for both liquids and solids, nonacid regurgitation of undigested food, respiratory symptoms (such as pneumonia, repeated aspiration, and nocturnal cough), heartburn, and weight loss.^[6,7]^ Furthermore, esophageal cancer risk is higher in those with achalasia.^[8]^ Achalasia is diagnosed by clinical suspicion, esophageal manometry, barium swallow, radiography, and upper gastrointestinal endoscopy.^[9]^ As will be explained below, there are a number of treatment options for achalasia. Sadly, none of them offer a permanent cure for the condition, and all of the ones that are currently available are meant to be palliative. Pharmaceuticals, botulinum toxin injections (BTX), pneumatic dilatation (PD), laparoscopic Heller myotomies (HMs), peroral endoscopic myotomies (POEMs), and esophagectomy for end-stage achalasia are among the treatments for achalasia.^[7,10]^ The initial attempt to treat esophageal achalasia was PD. Perforation is the most serious consequence that turned up.^[11]^ Albeit, PD does not require specialized training and is reasonably simple and inexpensive to perform without severe gastroesophageal reflux disease (GERD) occurrence.^[6,7]^ On the other hand, BTX is a straightforward endoscopic technique that can be repeated with ease, is conducted under direct view, and has minimal side effects or difficulties.^[12,13]^ Unfortunately, symptoms frequently return and necessitate repeated shots.^[14]^Meanwhile, HM is regarded as the gold standard in the treatment of achalasia because it can offer patients better and more durable symptom alleviation; nevertheless, older individuals and patients with numerous comorbidities are not candidates for HM, owing the fact that HM is an invasive, rather pricey surgery that needs general anesthesia.^[6,7,15–17]^But eventually, a minimally invasive approach for treating achalasia, POEM, has acquired global recognition.^[18,19]^ Compared to surgery, it is less intrusive and may result in reduced morbidity, a faster recovery, improved cosmesis, a longer myotomy, and the avoidance of thoracic and abdominal esophageal dissection.^[20]^

Although several meta-analyses have assessed the efficacy and safety of achalasia treatments, notable methodological flaws limit direct comparisons. These include inconsistencies in outcome definitions, follow-up durations, and statistical approaches. For instance, treatment success has been evaluated using various timeframes and measures; some studies rely on patient-reported dysphagia or symptom recurrence^[20,21]^ while others use composite scores like the Eckardt score.^[22,23]^ Similarly, the assessment of complications, particularly GERD, varies between endoscopic findings and patient-reported outcomes.^[22,24]^ Additionally, different statistical methods, such as risk differences, odds ratios (ORs), and standard mean differences, complicate interpretation. Recognizing these methodological and analytical flaws, this review aims to standardize outcome definitions, harmonize statistical methods, and evaluate the quality of existing evidence, offering clearer insights into treatment efficacy and guiding future research for more reliable findings.

## 2. Methodology

### 2.1. Setting

The primary goal of this study is to summarize the available literature regarding the meta-analyses conducted to assess the effectiveness and complications of different achalasia treatment options. This study was conducted based on the Preferred Reporting Items for Systematic Reviews and Meta-Analysis (PRISMA) criteria, ensuring that we report our findings using a thorough and organized methodology. The study protocol has been prospectively registered with PROSPERO under registration number CRD42024547223 to ensure transparent reporting and reduce sources of bias. This umbrella review on meta-analysis is based on previously published studies and does not involve new primary data collection. As such, ethical approval was not required for this study.

### 2.2. Search strategy

A comprehensive literature search was employed across 5 international databases,including PubMed, Scopus, Web of Science, Embase, and the Cochrane library. Our search extended from the inception of the databases up to August 20, 2025 and incorporated specific keywords such as “esophageal achalasia,” “Achalasia,” “cardiospasm,” “Megaesophagus,” “heller myotomy,” “laparoscopic heller myotomy,” “laparoscopic myotomy,” “myotomy,” “peroral endoscopic myotomy,” “pneumatic balloon dilation,” “endoscopic balloon dilatation,” “botulinum toxins,” “systematic review,” “meta-analysis,” and “meta-analyses.” We also searched the references of the included studies to identify other potentially relevant articles (Table S1, Supplemental Digital Content, https://links.lww.com/MD/Q643).

### 2.3. Inclusion and exclusion criteria

Prior to starting data screening, we developed a predetermined set of inclusion criteria based on the PICO framework. Study type: included articles must be meta-analysis studies. Population: adult individuals diagnosed with achalasia, regardless of disease subtype, etiology, or history of prior treatment. Intervention: POEM, HM, PD, or BTX. Comparative group: studies compared at least 2 mentioned treatment options together. Outcome: success or failure of the treatment (based on inadequate symptom management, need for reintervention, or relapse based on measurable scores such as Eckardt score), postoperative gastroesophageal reflux (based on pH-monitoring, esophago-gastro-duodenoscopy, patient reported symptoms, and GERD score on international questionnaires), operative time, length of myotomy, overall complication rate, major and minor complication rate based on Clavien-Dindo classification, rate of perforation, and length of hospital stay. We considered meta-analysis studies that analyzed only original papers comparing a minimum of 2 treatment alternatives in our systematic review. Excluded from consideration were meta-analyses that comprised papers of a single treatment strategy and were considered as part of their final analysis. Considering that most clinical trials and case control studies first match the groups’ baseline characteristics, we were able to minimize potential bias between the comparison groups. Additionally, studies focused on the child population or papers written in languages other than English were not included.

### 2.4. Study selection

Three experienced reviewers (NL, AH, M-HK) independently screened all search results based on predefined inclusion and exclusion criteria. The first step of data screening was based on title, and abstract and after excluding irrelevant results, the chosen papers went on full-text screening. Then the same 3 reviewers evaluated the full-texts of potentially relevant articles. Any discrepancies and disagreements in judgment between reviewers were resolved via discussion.

### 2.5. Data extraction

Finally, the fundamental characteristics of the selected studies were extracted by 3 reviewers independently (NL, AH, M-HK). Initial information, including: the first author’s name, year of publication, the country research was conducted in, the journal’s name, funding status, databases, and the date of search, number of included articles, sample size, and the main findings of the research paper, was inserted in Microsoft Excel tables.

### 2.6. Quality assessment

The methodological quality of the included studies was assessed using the AIMSTAR 2 grading system, which consists of 16 questions and 7 critical items including: prior written protocol, comprehensive literature search, justification of excluded articles, proper analytic method, risk of bias assessment of each study and its impact on the results, and adequate investigation of publication bias^[25]^ (Table S2, Supplemental Digital Content, https://links.lww.com/MD/Q643). These criteria were used to provide a score to each study, and the average score was used to assess the overall quality of the investigations. Three independent reviewers carried out the quality assessment, and disagreements were settled by consensus discussion (NL, EA-S, M-HK).

### 2.7. Statistical analysis

To establish a unified standard unit in each subject and to enable the demonstration of trends as well as the comparison of results from different meta-analysis studies, the Comprehensive Meta-Analyses (CMA) software version 3 (Biostat, Inc., Englewood) was used to analyze different treatment options for achalasia. The final results were presented using ORs with confidence intervals (CIs) for binary (dichotomous) variables. For continuous variables, the standard difference in means was calculated. In cases where direct conversion of effect sizes was not feasible, the original data from the studies included in the meta-analyses were used to generate new effect sizes, as well as to verify the accuracy of the analysis.

## 3. Result

### 3.1. Study selection

**Figure 1.**
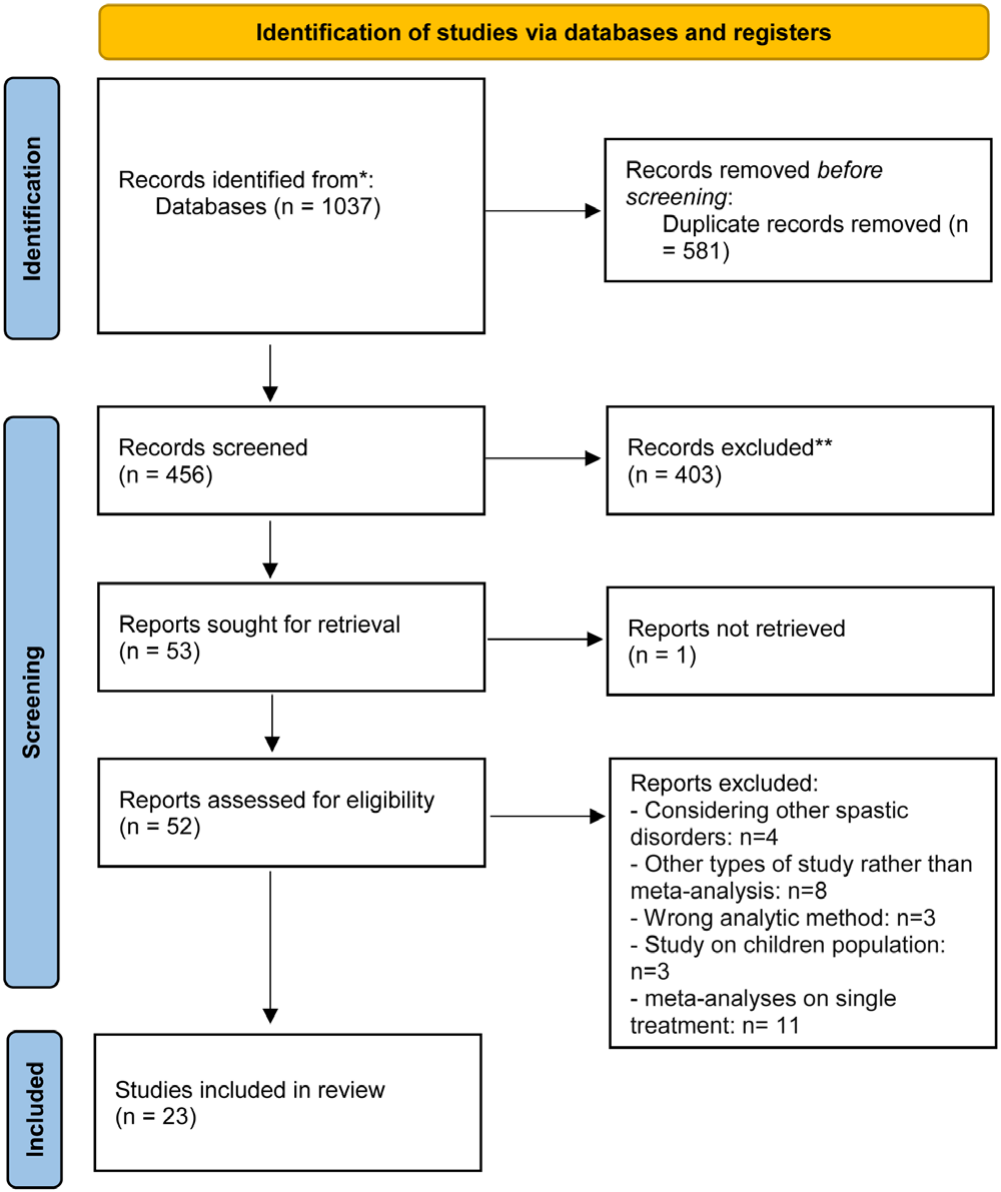
PRISMA flow diagram of study selection process. PRISMA = Preferred Reporting Items for Systematic Reviews and Meta-Analysis.

The search strategy initially identified a total of 1037 articles. After removing 581 duplicate studies, 456 articles were assessed based on the title and abstract. Following this initial screening, 52 studies underwent for further evaluation and finally 23 meta-analyses were selected in this study (Fig. 1). The characteristics of the included studies is presented in Table 1.

**Table 1:** Characteristics of included studies.

References	Journal	Country	Databases searched	Date of the search	Status of funding	Treatments	Number of studies	Sample size	Outcomes	Quality
Malik et al^[26]^	*Indian Journal of Gastroenterology*	USA	PubMed, Cochrane, Scopus, Web of Science, and Embase	NR	NR	HM vs PD	10	894	Success rate at 3 m, 1 y, 2 y, and 5 y, adverse events, GERD, perforation, relapse	Critically low
Ma et al^[27]^	*Surgical Endoscopy*	Canada	Embase, Medline, Web of Science, and CENTRAL	Jan 2024	Funded	POEM vs HM	9	1099	Success rate in follow-up periods of ≥24 m, operative duration, hospital stay, complications, GERD	Low
Sobral et al^[23]^	*Esophagus*	Portugal	PubMed, Web of Science, and Cochrane Library	Dec 2022	Funded	POEM vs HM	20	5139	GERD, success rate, postop Eckardt score, operative duration, hospital stay, complications	Low
So Taa Kum et al^[28]^	*Endoscopy International Open*	Brazil	Medline, Embase, Cochrane Library, and Clinicaltrials.gov	Nov 2024	NR	POEM vs HM	34	14,125	Early and late GERD, success rate, major adverse events, hospital stay, procedure time	Low
Baniya et al^[29]^	*Clinical and Experimental Gastroenterology*	USA	Pubmed, Cochrane, Scopus	2016	NR*	HM† vs PD‡	5	437	Success rate at 3 m, 1 y, and 5 y	Critically low
Bonifácio et al^[5]^	*Diseases of the Esophagus*	Brazil	Medline, Scopus, Latin-American, Caribbean Health Sciences Literature, Brazilian Virtual Library of Health, Cochrane	Jun 2018	NR	HM vs PD	4	404	Success rate at 2 y and 5 y, GERD, perforation	High
Cheng et al^[30]^	*Medicine*	China	Pubmed, Cochrane, Embase	Mar 2016	NR	HM vs PD	5	373	Success rate at 3 m, 1 y, 2 y, and 5 y, complications, GERD	High
Illés et al^[31]^	*Gastrointestinal Liver Disease*	Hungary	PubMed, Embase, Cochrane	Dec 2015	NR	HM vs PD	8	749	Success rate at 1 y, GERD, perforation,	Low
Wang et al^[32]^	*World Journal of Gastroenterology*	China	Chinese Biomedical, Chinese scientific Journals	Mar 2008	NR	HM vs PD	2	76	Success rate	Critically low
Wang et al^[32]^	*World Journal of Gastroenterology*	China	Chinese Biomedical, Chinese scientific Journals	Mar 2008	NR	PD vs BTX§	7	231	Success rate, recurrence rate	Critically low
Wang et al^[33]^	*Digestive Diseases and Sciences*	China	Medline, Embase, Cochrane, LILACS Latin American, Caribbean Health Science Literature, and Science Citation Index Expanded	Mar 2008	NR	HM vs PD	2	81	Success rate	Low
Wang et al^[33]^	*Digestive Diseases and Sciences*	China	Medline, Embase, Cochrane, LILACS Latin American, Caribbean Health Science Literature, and Science Citation Index Expanded	Mar 2008	NR	PD vs BTX	5	155	Success rate, recurrence, complication	Low
Yaghoobi et al^[34]^	*Gastrointestinal Endoscopy*	Canada	Medline, Embase, Cochrane, WOS	Mar 2012	None	HM vs PD	3	346	Success rate at 1 y, major complications	High
Zhang et al^[35]^	*International Journal of Clinical and Experimental Medicine*	China	PubMed, Embase, Cochrane, WanFang	Jul 2018	None	HM vs PD	8	236	Short term and long-term success rate, complication	High
Zhang et al^[35]^	*International Journal of Clinical and Experimental Medicine*	China	PubMed, WanFang, Embase, Cochrane	Jul 2018	None	PD vs BTX	8	386	Short term and long-term success rate, recurrence, complication	High
Dirks et al^[20]^	*Surgical Endoscopy*	USA	Medline, Embase, Cochrane, TRIP	Dec 2019	None	POEM∥ vs HM	20	1904	Patient-reported dysphagia, Eckardt score, patient-reported reflux, objective measure of GERD	Low
Dirks et al^[20]^	*Surgical Endoscopy*	USA	Medline, TRIP, Embase, Cochrane	Dec 2019	None	POEM vs PD	7	619	Eckardt scores, patient-reported reflux, recurrence	Low
Kum et al^[36]^	*Cureus*	Brazil	Medline, Scopus, Embase, Cochrane	Sep 2022	None	POEM vs HM	29	13,643	Early & late endoscopic findings of GERD, success rate, operative duration, hospital stay, major adverse events	Low
Kum et al^[36]^	*Cureus*	Brazil	Medline, Embase, Cochrane, Scopus	Sep 2022	None	POEM vs PD	2	261	Early & late endoscopic findings of GERD, success rate, operative duration, hospital stay, major adverse events	Low
Marano et al^[22]^	*Medicine*	Italy	Medline, Cochrane, Ovid	Jan 2015	None	POEM & HM	7	486	Eckardt score reduction, operative time, hospital stay, postoperative pain score, analgesic medication dose, major & minor & overall complications, GERD	High
Martins et al^[37]^	*Arquivos de Gastroenterologia*	Brazil	Medline, Embase, Scopus, LILACS, BVS, Cochrane	Apr 2019	None	POEM & HM	12	893	Operative time, GERD, hospital stay, Eckardt score reduction	High
Park et al^[24]^	*Gastrointestinal Endoscopy*	Korea	Cochrane, Medline, Embase	Sep 2018	None	POEM & HM	15	1213	Postoperative Eckardt scores, length of myotomy, operation time, hospital stay, pH monitoring, reflux symptoms, erosive esophagitis on endoscopy	High
Wei et al^[21]^	*Journal of Laparoendoscopic and Advanced Surgical Techniques*	China	Pubmed, Cochrane, Embase	Dec 2013	None	POEM & HM	4	242	Major & minor & overall complications, perforation, GERD, recurrence, pain assessment and medication usage, operative time, hospital stay	Low
Zhang et al^[10]^	*Medicine*	China	Google Scholar, Embase, Medline, Cochrane	Dec 2014	Funded	POEM & HM	4	317	Length of myotomy, operation time, hospital stay, Eckardt scores, complications	High
Zhong et al^[38]^	*European Journal of Gastroenterology & Hepatology*	China	Embase, Pubmed, Cochrane	Sep 2019	Funded	POEM & PD	7	619	Success rate at 3, 6, 12, 24 m, Eckardt score, postoperative LESP, Eckardt score reduction, LESP reduction, GERD, complications	Moderate
Campos et al^[39]^	*Annals of Surgery*	USA	Medline	2006	Funded	PD vs BTX	7	261	Success rate	Critically low
Leyden et al^[40]^	*The Cochrane Library*	Ireland	Medline, Embase, Cochrane, WOS	Sep 2008	Funded	PD vs BTX	4	132	Success rate at initial, 6m, 1 y	Low
Leyden et al^[12]^	*The Cochrane Library*	Ireland	Medline, WOS, Embase, Cochrane	Apr 2014	Funded	PD vs BTX	5	189	Success rate at initial, 6m, 1 y	Low

BTX = botulinum toxin injection, GERD = gastroesophageal reflux disease, HM = Heller myotomy, LESP = Lower Esophageal Sphincter Pressure, NR = not reported, PD = pneumatic dilatation, POEM = peroral endoscopic myotomy, WOS = Web of Science.

* Not reported.

† Heller myotomy.

‡ Pneumatic dilation.

§ Botulinum toxin injection.

∥ Peroral endoscopic myotomy.

### 3.2. POEM versus HM

#### 3.2.1. Studies characteristics and quality assessment

**Figure 2.**
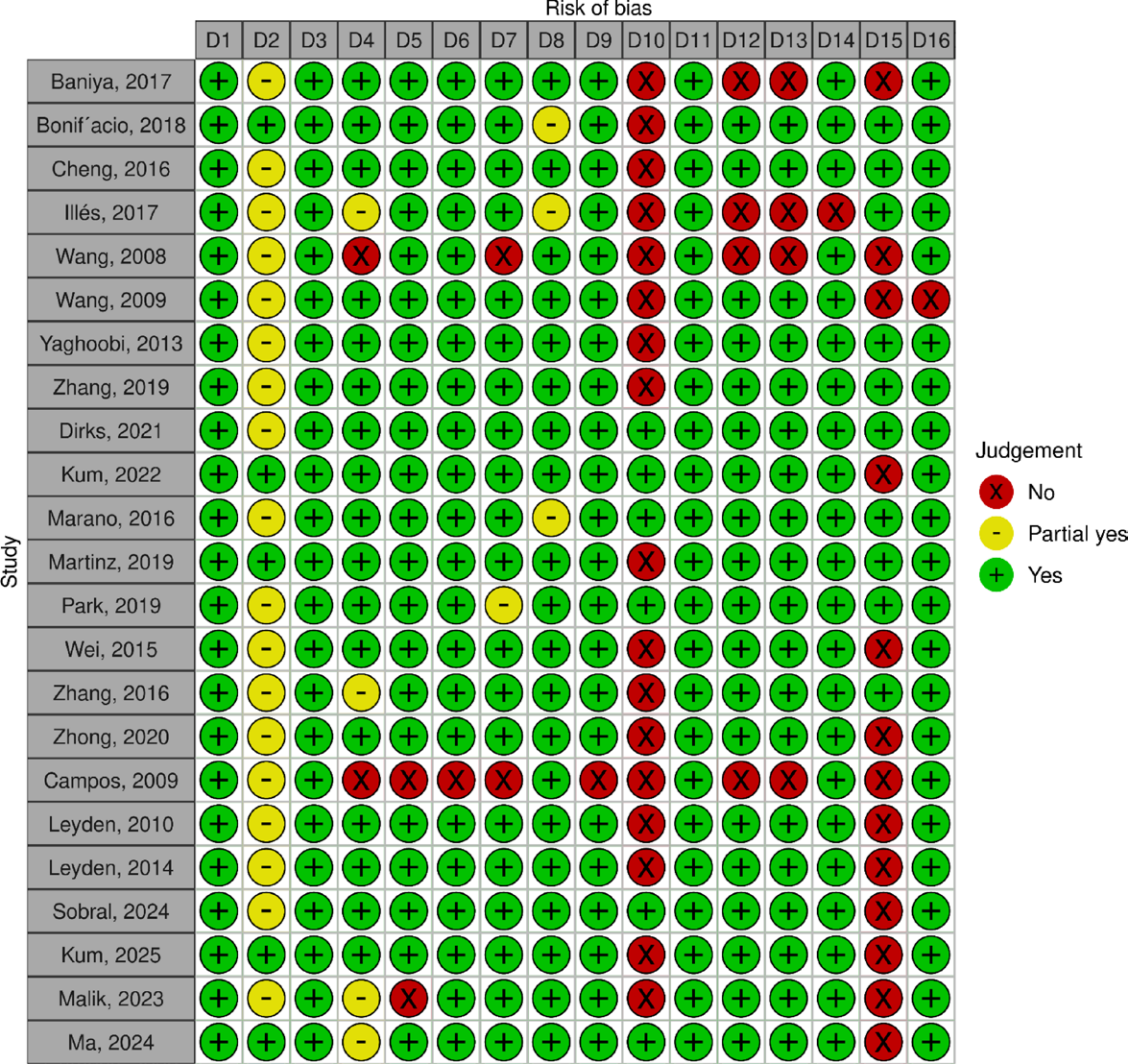
Quality assessment of included studies based on AMSTAR2 checklist. D1* to D16* correspond to the individual questions from the AMSTAR2 checklist. The graph was generated via Robvis (visualization tool).^[41]^ Green circles (+) indicate adequately reported items (low risk of bias); yellow circles (−) indicate partially reported or unclear items (moderate risk of bias); and red circles (×) indicate items not reported or inadequately described (high risk of bias).

Among the 10 meta-analyses comparing POEM and HM,^[10,20–24,27,28,36,37]^ 4 studies (Martins et al (2020), Park et al (2019), Marano et al (2016), and Zhang et al (2016)) were rated as high quality, while 5 studies (So Taa Kum et al (2025), Ma et al (2025), Sobral et al (2024), Kum et al (2022), and Dirks et al (2021)) were rated as low quality, and 1 study (Wei (2015)), was rated as critically low (Fig. 2).

The meta-analysis by So Taa Kum et al (2025), which included 32 observational studies and 2 randomized clinical trials (RCTs), provides the most recent information and utilizes the latest methodology for assessing GERD, as defined by the Lyon Consensus 2.0.^[28]^ Sobral et al (2024) analyzed data from a large sample size of 5139 participants,^[23]^ while Ma et al (2025) contributed valuable insights by comparing the long-term outcomes of POEM and HM.^[27]^ However, none of these studies performed statistical tests to assess the presence of publication bias or its potential effect on the results.

On the other hand, the common limitation of the older high-quality studies is the lack of protocol registration and the smaller sample sizes. However, we tried to balance and report data that is supported by high-quality studies, alongside the more reliable data based on larger sample sizes, particularly from the most recent publications.

#### 3.2.2. Clinical success rate

**Figure 3.**
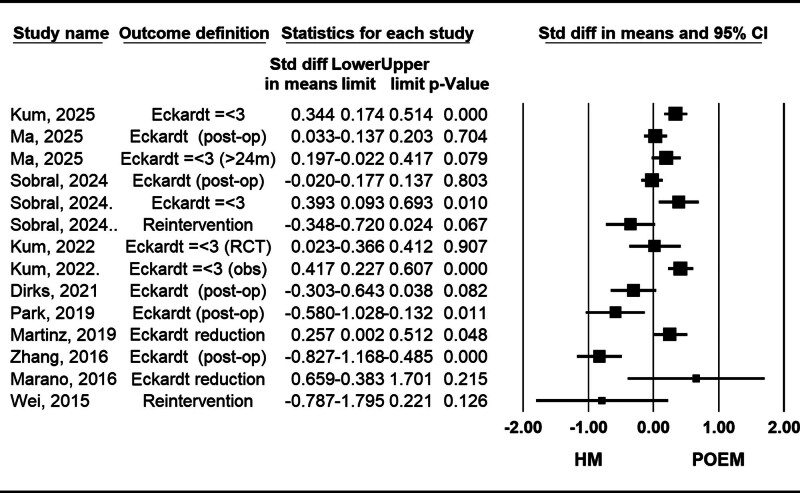
Forest plot of studies comparing clinical success rate between POEM (peroral endoscopic myotomy) and HM (Heller myotomy). The higher number of patients with an Eckardt score ≤3, higher score reduction, lower postoperative Eckardt score, and lower reintervention rate favor POEM. CI = confidence interval, obs = observational studies, RCT = randomized clinical trial.

Meta-analyses have explored the differences in clinical success rates between POEM and HM using various methodologies. According to the most recent evidence and the largest patient populations, So Taa Kum et al (2025) and Sobral et al (2024) found that POEM demonstrated superior efficacy compared to HM in achieving an Eckardt score ≤3, with significant homogeneity among the included studies.^[23,28]^ In contrast, the study by Ma et al (2025) showed no superiority of either treatment in terms of success rates during longer follow-up periods.^[27]^ Studies with higher AIMSTAR 2 quality scores, such as Martins et al (2020) and Park et al (2019), also suggested that POEM offers better short-term success^[24,37]^ (Fig. 3).

#### 3.2.3. Gastroesophageal reflux

**Figure 4.**
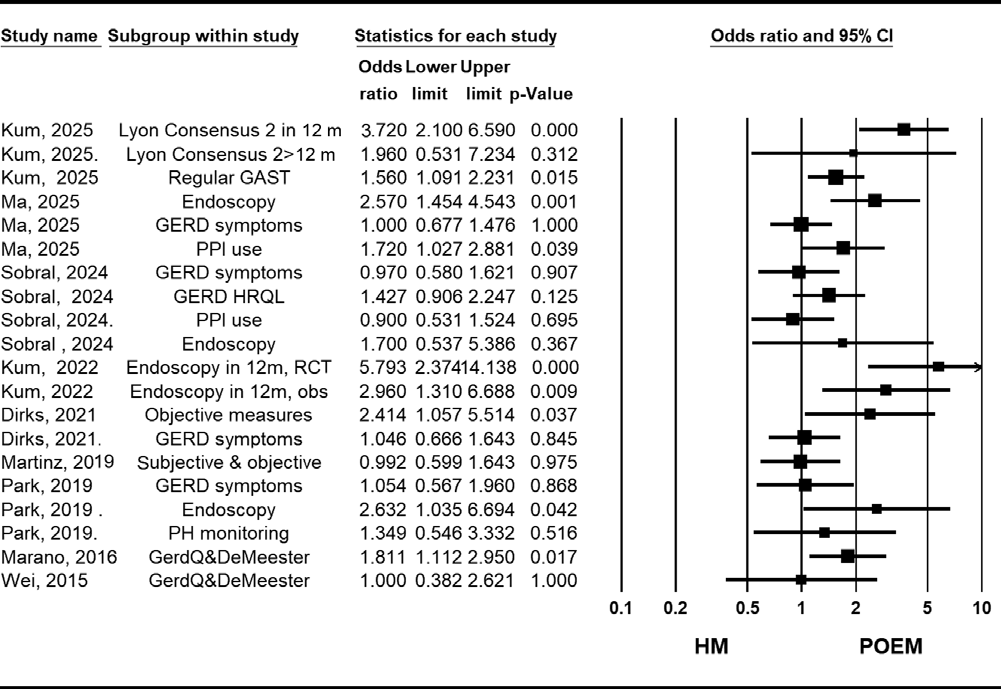
Forest plot of studies comparing gastroesophageal reflux rate between POEM (peroral endoscopic myotomy) and HM (Heller myotomy). 12m = 12 months, CI = confidence interval, GAST = gastric acid suppression therapy, GERD = gastroesophageal reflux disease, HRQL = health related quality of life, obs = observational studies, RCT = randomized clinical trial.

Nine studies compared the incidence of GERD between patients undergoing POEM and HM, using methods such as patient-reported symptoms, proton pump inhibitor use, pH monitoring, and endoscopy, reporting controversial data. However, the study by So Taa Kum et al (2025) used the most updated method of assessing GERD based on the Lyon Consensus 2.0.^[28]^ They found a higher GERD incidence following POEM in early evaluations, but the difference diminished over time, likely due to increased gastric acid suppressors use in the POEM group.^[28]^ Similarly, Ma et al (2025) demonstrated higher rates of proton pump inhibitor use in the POEM group.^[27]^ Other meta-analyses with fewer included studies used outdated GERD assessment methods, limiting their applicability (Fig. 4).

#### 3.2.4. Complications

**Figure 5.**
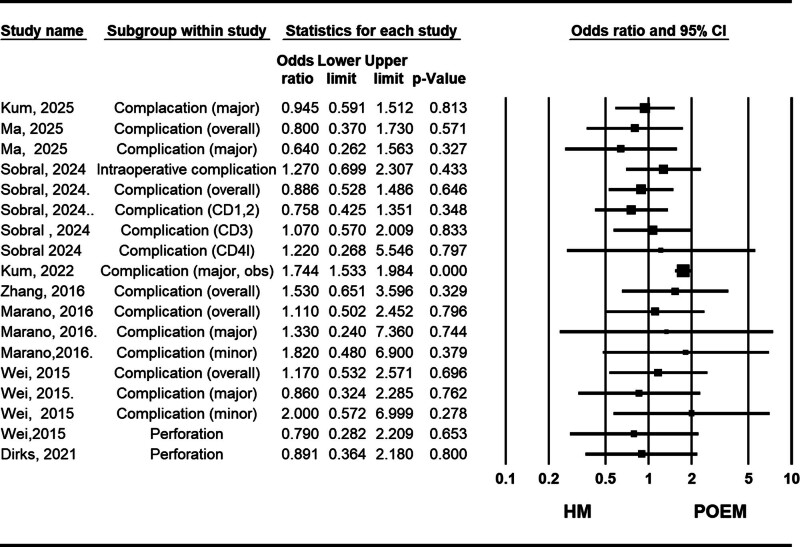
Forest plot of studies comparing complication rate between POEM (peroral endoscopic myotomy) and HM (Heller myotomy). CD = Clavien–Dindo classification, CI = confidence interval, obs = observational studies.

Complications were commonly categorized into major and minor groups based on the Clavien-Dindo classification in the included studies. Among the high-quality studies,^[10,22,24,37]^ the work by Marano et al (2016)^[22]^ provided the most comprehensive description of complication differences between the POEM and HM groups. Across all 3 reporting methods (overall, major, and minor complications), the POEM group exhibited a safety profile comparable to that of the HM group. These findings are consistent with the results from recent meta-analyses with larger sample sizes, including those by So Taa Kum et al (2025), Ma et al (2025), and Sobral et al (2024)^[23,27,28]^ (Fig. 5).

#### 3.2.5. Operation time

**Figure 6.**
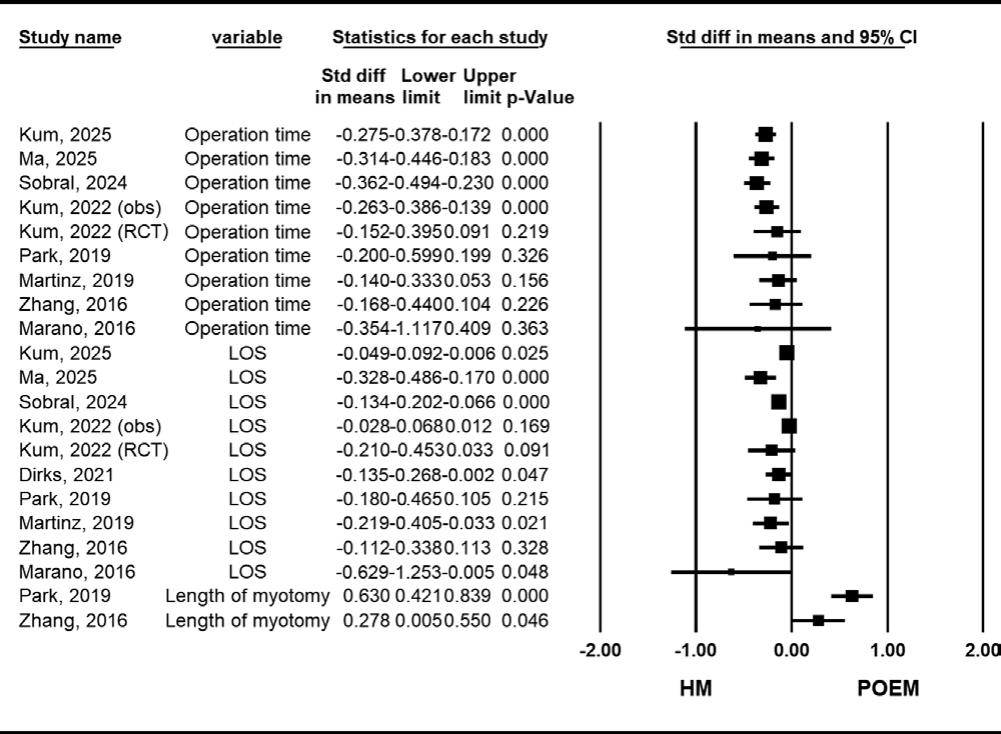
Forest plot of studies comparing operation time, length of hospital stay (LOS), and length of myotomy between POEM (peroral endoscopic myotomy) and HM (Heller myotomy). CI = confidence interval, obs = observational studies, RCT = randomized clinical trial.

Nine meta-analyses compared operative time between patients undergoing POEM and those receiving HM. Among the high-quality studies, including Park et al (2019), Zhang et al (2016), Martins et al (2020), and Marano et al (2016), no significant difference in operative time was observed between the 2 procedures.^[10,22,24,37]^ However, these studies reported high heterogeneity, which was primarily attributed to the learning curve associated with the POEM technique. So Taa Kum et al (2025), which included more adequate data from 20 studies on procedure time, demonstrated that POEM is a more time-efficient surgical approach for the treatment of achalasia^[28]^ (Fig. 6).

#### 3.2.6. Length of myotomy

Two high quality meta-analysis studies of Park et al (2019) and Zhang et al (2016) specifically evaluated the length of the myotomy procedure between the POEM and HM groups.^[10,24]^ The results demonstrated that the POEM approach was associated with a longer myotomy length compared to the HM procedure. However, there is a lack of detailed information on this topic in the latest meta-analyses (Fig. 6).

#### 3.2.7. Length of hospital stay

Nine meta-analyses compared length of hospital stay between patients undergoing POEM and HM. Although rated as low quality, Dirks et al (2021) made the most effort to address heterogeneity by stratifying based on study quality, age group, country, and publication date.^[20]^ Subgrouping studies based in the United States, Dirks et al (2021) found a significantly shorter hospital stay in the POEM group, with substantially reduced heterogeneity. This result was further supported by the recent meta-analyses by So Taa Kum et al (2025), Ma et al (2025), and Sobral et al (2024)^[23,27,28]^ (Fig. 6).

#### 3.2.8. Publication bias assessment

In the studies comparing POEM and HM, a notable deficiency in publication bias assessment was observed, especially in the recent meta-analyses that incorporated the latest available data. To mitigate this gap, we referenced the data comparing clinical success rates presented in So Taa Kum et al (2025) to evaluate publication bias.^[28]^ As shown in Figure S1, Supplemental Digital Content, https://links.lww.com/MD/Q643, the trim and fill method imputed 2 studies on the left side. However, the adjusted values considering 2 additional studies (OR = 1.75, 95% CI: 1.30–2.37) did not differ significantly from the observed values (OR = 1.87, 95% CI: 1.37–2.54). In addition, the Egger (*P* = .58) and Begg (*P* = .67) tests did not support the presence of publication bias.

### 3.3. POEM versus PD

#### 3.3.1. Studies characteristics and quality assessment

Among the 2 meta-analyses comparing the efficacy of POEM and PD, the 2020 study by Zhong et al was rated as moderate quality.^[38]^ A limitation of this study was its inadequate assessment of heterogeneity. However, they performed a subgroup analysis based on achalasia subtypes in their evaluation of clinical success. The second meta-analysis, Dirks et al. (2021), included 28 studies, 8 of which directly compared POEM and PD.^[20]^ Although rated as lower quality due to the lack of formal publication bias assessment, it employed rigorous methods, including subgroup analyses based on study quality and sensitivity analyses to address heterogeneity. The absence of publication bias assessment remains a limitation, particularly given the small number of included studies (Fig. 2).

#### 3.3.2. Clinical success rate

**Figure 7.**
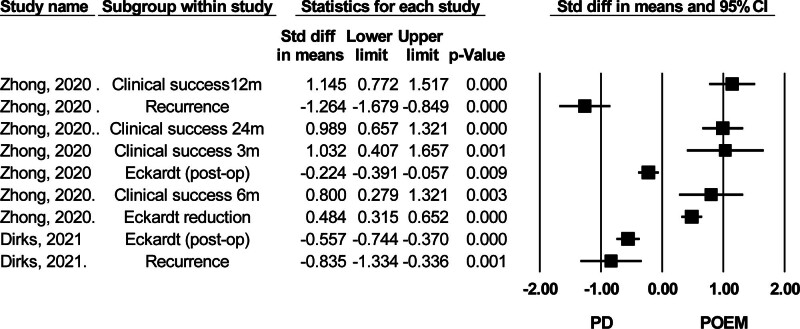
Forest plot of studies comparing clinical success rate between POEM (peroral endoscopic myotomy) and PD (pneumatic dilation). Higher clinical success in different durations, higher Eckardt score reduction, lower postoperative Eckardt score, and lower recurrence rate favor POEM. CI = confidence interval.

As shown in Figure 7, POEM demonstrated superior efficacy compared to PD in achieving an Eckardt score ≤3 across various follow-up durations, ranging from 3 months to 2 years. Also, POEM was associated with a significantly greater reduction in Eckardt scores from pre- to postoperative assessment and exhibited significantly lower postoperative scores.

#### 3.3.3. Reflux and other complications

**Figure 8.**
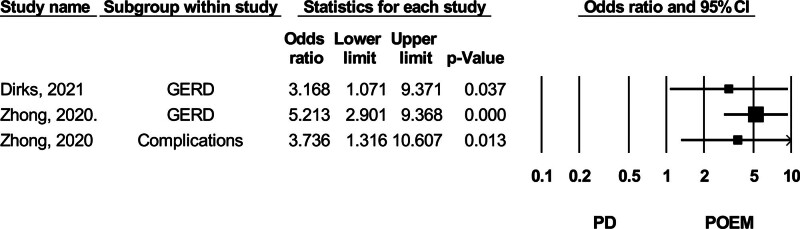
Forest plot of studies comparing GERD (gastroesophageal reflux disease) and other complications between POEM (peroral endoscopic myotomy) and PD (pneumatic dilation). CI = confidence interval.

The 2 studies evaluating GERD rates found a higher incidence in the POEM group compared to the PD group.^[20,38]^ Dirks et al (2021) reported data based on patient symptoms, while Zhong et al (2020), who reported nearly a 5-fold higher GERD rate in the POEM group, did not specify the assessment method.^[20]^ Zhong et al (2020) also reported complications, including subcutaneous emphysema, mucosal injuries, and bleeding, with POEM showing a higher risk of complications than PD^[38]^ (Fig. 8).

#### 3.3.4. Publication bias assessment

Although the use of funnel plots in meta-analyses with fewer than 10 included studies is limited, the study by Zhong et al (2020) assessed publication bias through visual inspection of the funnel plot across different outcomes and found that no publication bias can generally be considered.^[38]^

### 3.4. HM versus PD

#### 3.4.1. Studies characteristics and quality assessment

Among the 9 meta-analyses comparing HM and PD, the studies by Zhang et al (2019) (on 8 RCTs), Bonifácio et al (2019) (on 4 RCTs), Cheng et al (2017) (on 5 RCTs), and Yaghoobi et al (2013) (on 5 RCTs) were rated as high quality based on the AIMSTAR 2 checklist.^[5,30,34,35]^ Conversely, the studies by Illés et al (2017), Wang et al (2008), and Wang et al (2009) were assessed as low quality,^[31–33]^ while the meta-analyses by Malik et al (2024) and Baniya et al (2017) were rated critically low^[26,29]^ (Fig. 2).

Most of the evidence comparing outcomes between HM and PD comes from RCTs. The Malik et al (2024) meta-analysis, rated critically low, was criticized for incorrect interpretation of results, the absence of publication bias assessment, and failure to specify the exact search dates for databases. Nevertheless, it included the most recent data from 10 RCTs comparing these 2 approaches, and the correct analysis and results are provided here.^[26]^

#### 3.4.2. Clinical success rate

**Figure 9.**
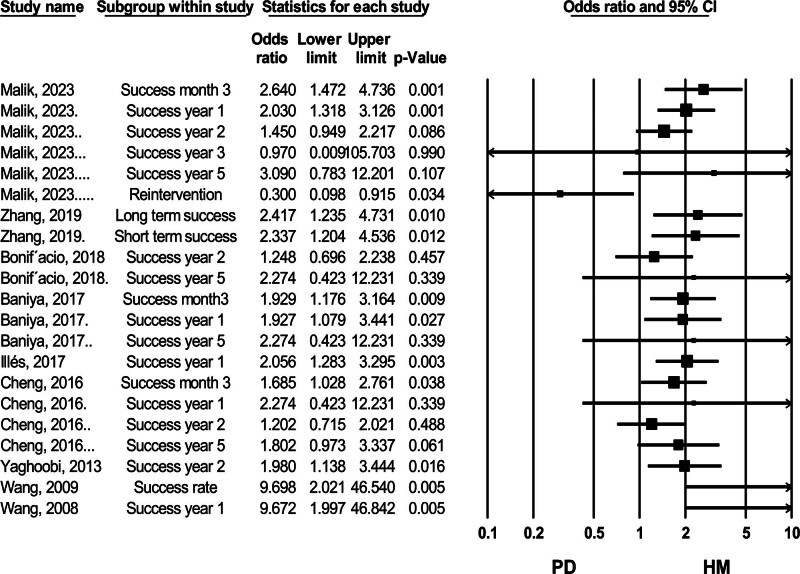
Forest plot of studies comparing success rate between HM (Heller myotomy) and PD (pneumatic dilation). CI = confidence interval.

Among the high-quality meta-analyses comparing the efficacy of HM and PD, the most recent study by Zhang et al (2019) demonstrated better efficacy for HM in both short-term and long-term follow-ups, although the specific timeframes were not clearly defined.^[35]^ In contrast, Bonifácio et al (2019) and Cheng et al (2017) found no statistically significant difference in success rates between HM and PD in the long-term follow-up^[5,30]^ (Fig. 9).

In addition to these high-quality studies, our analysis of the most recent data from Malik et al (2024) showed that HM had better efficacy in terms of remission rates at 3 months and 1 year. However, this benefit was not sustained in longer follow-up periods^[26]^ (Fig. 9).

#### 3.4.3. Gastroesophageal reflux

**Figure 10.**
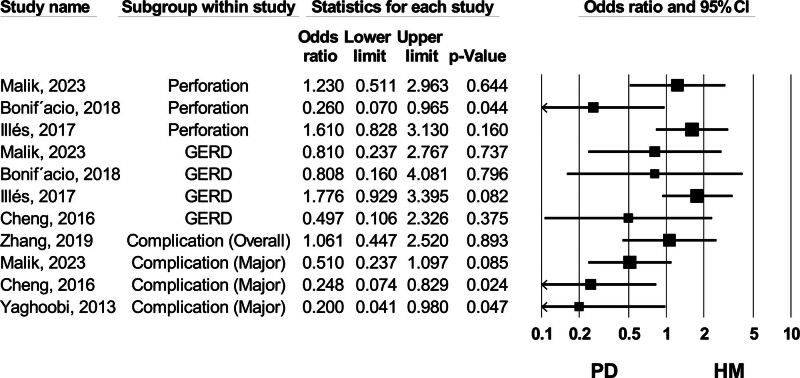
Forest plot of studies comparing GERD (gastroesophageal reflux disease), perforation, and complication rates between HM (Heller myotomy) and PD (pneumatic dilation). CI = confidence interval.

None of the included meta-analyses found a substantial difference in GERD rates between patients undergoing HM or PD^[5,30,31]^ (Fig. 10).

#### 3.4.4. Perforation

Perforation is defined as a complete rupture of the muscle layers in patients undergoing PD, whereas it is considered a rupture of the esophageal mucosa in patients undergoing HM. Three meta-analyses assessed the perforation rates between these 2 groups.^[5,26,31]^ Among these, the most recent data from Malik et al (2024), which included 7 RCTs, revealed no statistically significant difference between the 2 groups^[26]^ (Fig. 10).

#### 3.4.5. Complications

Four meta-analyses evaluated complications between 2 groups of patients treated with HM and PD.^[26,30,34,35]^ Based on the AMSTAR2 checklist, 3 studies (Zhang et al (2019), Cheng et al (2017), and Yaghoobi et al (2013)) were rated as high quality. However, there is a significant time gap between these studies and the lower-quality meta-analysis by Malik et al (2024), which reported no statistically meaningful difference between the 2 treatment methods based on data from 9 RCTs^[26]^ (Fig. 10).

#### 3.4.6. Publication bias assessment

The meta-analysis of Zhang et al (2019) on 8 RCTs reported no obvious publication bias in reporting success rate as the shape of the funnel plot appeared symmetrical.^[35]^

#### 3.4.7. PD versus BTX

**Figure 11.**
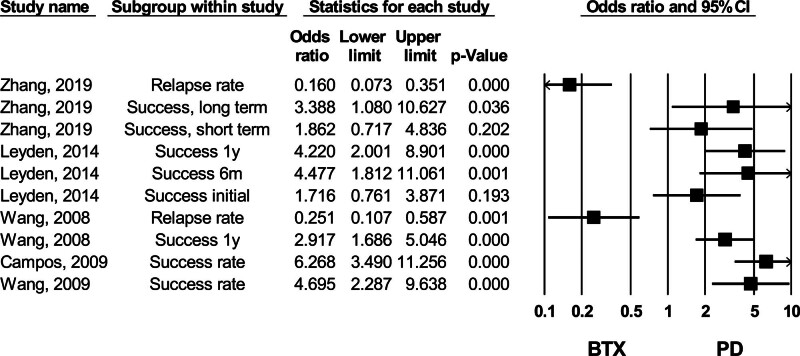
Forest plot of studies comparing success and relapse rate between PD (pneumatic dilation) and BTX (botulinum toxin injection). CI = confidence interval.

Of the 6 meta-analyses comparing PD and BTX injection, Zhang et al (2019) was deemed high quality according to the AMSTAR2 checklist.^[35]^ This study, conducted in 2019, reported that PD had better long-term success compared to BTX injection, with significantly lower relapse rates in the PD group.^[35]^ These findings were consistent across all 6 meta-analyses, as depicted in Figure 11.^[12,32,33,35,39,40]^ No studies assessed the publication bias through their analysis.

## 4. Discussion

This umbrella review of meta-analysis studies compares the efficacy and safety of the most recognized treatments for achalasia: POEM, HM, PD, and BTX. Based on disparate quality evidence, POEM and HM demonstrate comparable efficacy and similar safety profiles, with both therapies showing better efficacy than PD, reinforcing their position as the preferred treatment options for achalasia.

### 4.1. Short-term efficacy: POEM vs HM

In terms of short-term efficacy, studies consistently confirm that POEM results in better clinical success in the early postoperative period, particularly in terms of symptom relief and dysphagia reduction compared to HM.^[23,24,37]^ However, it is important to note that long-term efficacy between POEM and HM is generally comparable, with both treatments providing sustained relief over time.^[27]^ This is consistent with findings from Ma et al (2025), the only meta-analysis where follow-ups beyond 24 months showed that HM maintained its efficacy over time, offering long-term symptom relief similar to POEM.^[27]^

### 4.2. Type-specific considerations

Meta-analysis studies did not specifically analyze outcomes for different achalasia subtypes. However, based on the American College of Gastroenterology’s 2020 guidelines, POEM is recommended as the treatment of choice for type III achalasia due to its ability to extend the myotomy into hypercontractile esophageal segments, which is crucial for this subtype.^[42]^ This recommendation is consistent with the 2017 American Gastroenterological Association andAmerican Society for Gastrointestinal Endoscopy 2020 guidelines, which recognize POEM as the most effective treatment for type III achalasia. POEM ability to address hypercontractility in these patients gives it a distinct advantage in treating spastic esophageal segments, which is a hallmark of type III.^[43]^

In contrast, HM remains the more suitable treatment for sigmoid achalasia, where the esophagus is more dilated and requires a more standardized myotomy.^[43,44]^ Studies suggest that HM is particularly effective in sigmoid achalasia, especially when combined with fundoplication to manage GERD.^[43]^ The combination of myotomy and fundoplication helps control both the esophageal pressure and the reflux risk that is common after myotomy, making HM a reliable choice for patients with advanced disease.^[44]^

### 4.3. Comparison with PD and BTX

When comparing PD and BTX to POEM and HM, it is evident that these therapies do not meet the same expectations for long-term therapeutic success. PD has been shown to have lower long-term efficacy, with higher recurrence rates and reinterventions required compared to POEM and HM.^[20,35,38]^ While PD is a noninvasive and cost-effective option, it is probably better suited for patients with mild forms of achalasia or those who are not ideal candidates for more invasive procedures. Similarly, BTX is often used for patients not suited for surgery, but its long-term efficacy remains limited, with recurrent symptoms and the need for repeated interventions being common. BTX and PD are therefore primarily viable for patients with comorbidities or those who are poor surgical candidates.^[45,46]^ As emphasized in the literature, patient preferences, cost, and comorbidities should drive the selection of treatment, and both PD and BTX serve as noninvasive options in such cases.

### 4.4. Gastroesophageal reflux disease

GERD remains a significant concern with POEM, particularly in the early postoperative phase. As noted in Section 3, POEM is associated with higher rates of GERD compared to HM in the early evaluations, primarily due to the absence of an anti-reflux procedure in POEM.^[28]^ However, this issue tends to diminish over time, with long-term GERD outcomes showing no significant difference between POEM and HM.^[28]^ It is important to note that POEM-related GERD can be effectively managed with PPIs, and long-term follow-up suggests that GERD rates stabilize in the months following the procedure.^[23,28]^

Traditional diagnostic methods for GERD, such as pH monitoring and symptom questionnaires, are often insufficient in the context of achalasia, where achalasia symptoms can mimic GERD.^[47,48]^ Lyon Consensus 2.0 emphasizes the need for modern diagnostic criteria to accurately distinguish GERD symptoms from achalasia-related symptoms.^[49]^ These updated diagnostic tools are essential for accurately assessing POEM-related GERD, as dysphagia and regurgitation in achalasia patients can often be mistaken for GERD.

### 4.5. Operative time, learning curve, and hospital stay

Recent meta-analyses have shown that POEM generally has a shorter operative time compared to HM.^[27,28]^ This difference has become more prominent in recent years, as learning curve factors have allowed endoscopists to become more skilled with the procedure over time, leading to increased efficiency in performing POEM.^[22,50]^

In contrast, HM tends to have more predictable and consistent operative times, as it is a well-established technique. However, POEM offers significant advantages in terms of hospital stay, as it is a minimally invasive technique, leading to shorter recovery times compared to HM, which involves a more invasive approach and typically requires a longer recovery period.^[51,52]^

Additionally, POEM allows for longer myotomies, which is particularly crucial for type III achalasia, where extended myotomies are necessary to effectively treat hypercontractile segments of the esophagus.^[10,24]^

### 4.6. Study limitations and future research directions

Despite the promising findings, there are several limitations in the meta-analyses reviewed, as well as the original studies they included, that must be acknowledged. In the meta-analyses, the heterogeneity across studies (due to variations in sample size, study quality, follow-up duration, and patient characteristics) complicates the interpretation of results. Additionally, the absence of subgroup-specific analyses for different achalasia subtypes, specifically type III and sigmoid achalasia, makes it difficult to assess how treatments perform across specific patient populations. The limited follow-up durations in many studies also hinder the evaluation of the long-term efficacy of these treatments, particularly for a chronic condition like achalasia. Furthermore, the absence of publication bias assessment and the failure to disclose primary research funding sources in many studies raises concerns about funding bias and publication bias, which may skew the representation of treatment efficacy. In the original studies themselves, methodological differences (particularly in how GERD, adverse events, and co-interventions were defined) limit comparability across studies. For instance, prior interventions such as recurrent balloon dilation may complicate POEM procedures due to lower esophageal sphincter muscle layer adhesions, which can impact the effectiveness of POEM or HM.^[35]^

These issues further contribute to the challenges of drawing clear conclusions from the data. Future research should address these limitations by including longer follow-up periods, subgroup-specific analyses, standardizing methodologies, and ensuring transparency in funding sources to improve the reliability of the findings.

## 5. Conclusion

In conclusion, both POEM and HM have proven to be effective treatment options for achalasia, each with its own advantages depending on patient needs and disease progression. While POEM offers promising short-term outcomes and quicker recovery, HM remains a well-established approach with reliable long-term results. GERD remains an important consideration in treatment planning, particularly with POEM, but it can generally be managed with appropriate medical interventions. Nonsurgical treatments, such as PD and BTX, continue to have a role in the management of patients who may not be suitable candidates for more invasive procedures. The selection of the most appropriate treatment should take into account patient-specific factors, disease severity, and the surgeon’s expertise. Future research, particularly with longer follow-up periods and more standardized methodologies, will help refine current treatment strategies and improve outcomes for patients with achalasia.

## Author contributions

**Conceptualization**: Negin Letafatkar, Fariborz Mansour-Ghanaei.

**Data curation**: Negin Letafatkar.

**Formal analysis**: Negin Letafatkar, Soheil Hassanipour.

**Investigation**: Negin Letafatkar, Arman Habibi.

**Methodology**: Negin Letafatkar, Arman Habibi, Fatemeh Mohammadyari, Mohammad-Hossein Keivanlou, Ehsan Amini-Salehi.

**Project administration**: Negin Letafatkar.

**Software**: Negin Letafatkar.

**Supervision**: Negin Letafatkar, Farahnaz Joukar, Soheil Hassanipour, Fariborz Mansour-Ghanaei.

**Validation**: Negin Letafatkar, Farahnaz Joukar, Mohammad-Hossein Keivanlou, Ehsan Amini-Salehi, Fariborz Mansour-Ghanaei.

**Writing – original draft**: Negin Letafatkar, Arman Habibi, Fatemeh Mohammadyari, Seyed-Kazem Hosseini-Ghaziani.

**Writing – review & editing**: Negin Letafatkar, Arman Habibi, Fatemeh Mohammadyari, Sara Nobakht, Mohammad-Hossein Keivanlou, Ehsan Amini-Salehi, Fariborz Mansour-Ghanaei.

## Supplementary Material
