# Supplementary material for: Comprehensive evaluation of current strategies in achalasia treatment: Insights from an umbrella review
Source: Medicine (Baltimore). 2026 May 12;104(49):e45885. doi: 10.1097/MD.0000000000045885 (PMC12689109; doi:10.1097/MD.0000000000045885)
Supplement: Supplementary file 1 [file medi-104-e45885-s001.docx]

| **PubMed** | ("esophageal achalasia"[MeSH Terms] OR "Achalasia"[Title/Abstract] OR "cardiospasm*"[Title/Abstract] OR "Megaesophagus"[Title/Abstract]) AND ("heller myotomy"[MeSH Terms] OR "pneumatic dilation"[Title/Abstract] OR "peroral endoscopic myotomy"[Title/Abstract] OR "pneumatic balloon dilation"[Title/Abstract] OR "laparoscopic heller myotomy"[Title/Abstract] OR "endoscopic balloon dilatation"[Title/Abstract] OR "botulinum toxins"[MeSH Terms] OR "laparoscopic myotomy"[Title/Abstract] OR "myotomy"[MeSH Terms]) AND ("systematic review"[Title/Abstract] OR "meta-analysis"[Title/Abstract] |
| --- | --- |
| **Scopus** | ( ( TITLE-ABS-KEY ( "esophageal achalasia" ) OR TITLE-ABS-KEY ( achalasia ) OR TITLE-ABS-KEY ( cardiospasm ) OR TITLE-ABS-KEY ( megaesophagus ) ) ) AND ( ( TITLE-ABS-KEY ( "systematic review" ) OR TITLE-ABS-KEY ( "meta-analysis" ) OR TITLE-ABS-KEY ( "meta analyses" ) ) ) AND ( ( TITLE-ABS-KEY ( "heller myotomy" ) OR TITLE-ABS-KEY ( "pneumatic dilation" ) OR TITLE-ABS-KEY ( "peroral endoscopic myotomy" ) OR TITLE-ABS-KEY ( "pneumatic balloon dilation" ) OR TITLE-ABS-KEY ( "laparoscopic heller myotomy" ) OR TITLE-ABS-KEY ( endoscopic AND balloon AND dilatation ) OR TITLE-ABS-KEY ( botulinum AND toxins ) OR TITLE-ABS-KEY ( laparoscopic AND myotomy ) OR TITLE-ABS-KEY ( myotomy ) ) ) |
| **Web of Science** | ("esophageal achalasia" OR "Achalasia" OR "cardiospasm" OR "Megaesophagus") AND ("heller myotomy" OR "pneumatic dilation" OR "peroral endoscopic myotomy" OR "pneumatic balloon dilation" OR "laparoscopic heller myotomy" OR "endoscopic balloon dilatation" OR "botulinum toxins" OR "laparoscopic myotomy" OR "myotomy") AND ("systematic review" OR "meta-analysis" OR "meta analyses") |
| **Embase** | ('esophageal achalasia'/exp OR achalasia:ti,ab OR cardiospasm*:ti,ab OR megaesophagus:ti,ab) AND  ('heller myotomy'/exp OR 'pneumatic dilation':ti,ab OR 'peroral endoscopic myotomy':ti,ab OR 'pneumatic balloon dilation':ti,ab OR 'laparoscopic heller myotomy':ti,ab OR 'endoscopic balloon dilatation':ti,ab OR 'botulinum toxin'/exp OR 'laparoscopic myotomy':ti,ab OR 'myotomy'/exp) AND  ('systematic review':ti,ab OR 'meta-analysis':ti,ab OR 'meta analyses':ti,ab) |
| **Cochrane library** | ("esophageal achalasia" OR "Achalasia" OR "cardiospasm" OR "Megaesophagus") AND ("heller myotomy" OR "pneumatic dilation" OR "peroral endoscopic myotomy" OR "pneumatic balloon dilation" OR "laparoscopic heller myotomy" OR "endoscopic balloon dilatation" OR "botulinum toxins" OR "laparoscopic myotomy" OR "myotomy") AND ("systematic review" OR "meta-analysis") |

**Supplementary Table 1**. Detailed search strategies used in each database (PubMed, Scopus, Web of Science, Embase, and Cochrane Library) for identifying relevant systematic reviews and meta-analyses related to treatment interventions for esophageal achalasia.

| Question 1 | Did the research questions and inclusion criteria for the review include the components of PICO? |
| --- | --- |
| Question 2 | Did the report of the review contain an explicit statement that the review methods were established prior to the conduct of the review and did the report justify any significant deviations from the protocol? |
| Question 3 | Did the review authors explain their selection of the study designs for inclusion in the review? |
| Question 4 | Did the review authors use a comprehensive literature search strategy? |
| Question 5 | Did the review authors perform study selection in duplicate? |
| Question 6 | Did the review authors perform data extraction in duplicate? |
| Question 7 | Did the review authors provide a list of excluded studies and justify the exclusions? |
| Question 8 | Did the review authors describe the included studies in adequate detail? |
| Question 9 | Did the review authors use a satisfactory technique for assessing the risk of bias (RoB) in individual studies that were included in the review? |
| Question 10 | Did the review authors report on the sources of funding for the studies included in the review? |
| Question 11 | If meta-analysis was performed did the review authors use appropriate methods for statistical combination of results? |
| Question 12 | If meta-analysis was performed, did the review authors assess the potential impact of RoB in individual studies on the results of the meta-analysis or other evidence synthesis? |
| Question 13 | Did the review authors account for RoB in individual studies when interpreting/ discussing the results of the review? |
| Question 14 | Did the review authors provide a satisfactory explanation for, and discussion of, any heterogeneity observed in the results of the review? |
| Question 15 | If they performed quantitative synthesis did the review authors carry out an adequate investigation of publication bias (small study bias) and discuss its likely impact on the results of the review? |
| Question 16 | Did the review authors report any potential sources of conflict of interest, including any funding they received for conducting the review? |

**Supplementary Table 2.** Full list of AMSTAR2 quality assessment questions used to evaluate the methodological quality of the included systematic reviews.

**Supplementary Figure 1: Funnel plot of studies comparing success rate between patients treated with POEM and HM.**
